# Supplementary material for: Neolithic introgression of IL23R-related protection against chronic inflammatory bowel diseases in modern Europeans
Source: eBioMedicine. 2025 Feb 8;113:105591. doi: 10.1016/j.ebiom.2025.105591 (PMC11849592; doi:10.1016/j.ebiom.2025.105591)
Supplement: Supplementary Figures S1–S6 [file mmc1.docx]

Supplementary Materials for:

**Neolithic introgression of *IL23R*-related protection against chronic inflammatory bowel diseases in modern Europeans**

**Authors**

Ben Krause-Kyora^1^, Nicolas Antonio da Silva^1^, Elif Kaplan^1^, Daniel Kolbe^1^, Archaeological Civilization Disease Consortium (ACDC)^*^, Inken Wohlers^2^, Hauke Busch^3^, David Ellinghaus^1^, Amke Caliebe^4^, Efe Sezgin^5^, Almut Nebel^1^, Stefan Schreiber^1,6^

**This file includes:**

Supplementary Figures S1 - S6

**
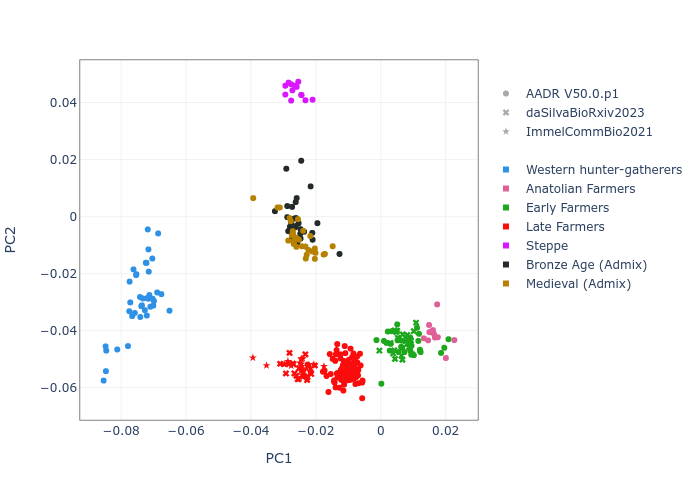
**

**Supplementary Figure S1.** Principal component analysis (PCA) of ancient samples used in this study. The ancient samples are projected on top of the PCs calculated using 64 modern West Eurasian populations from the Human Origins panel (not shown). Colours indicate distinct population genetic groups.

**
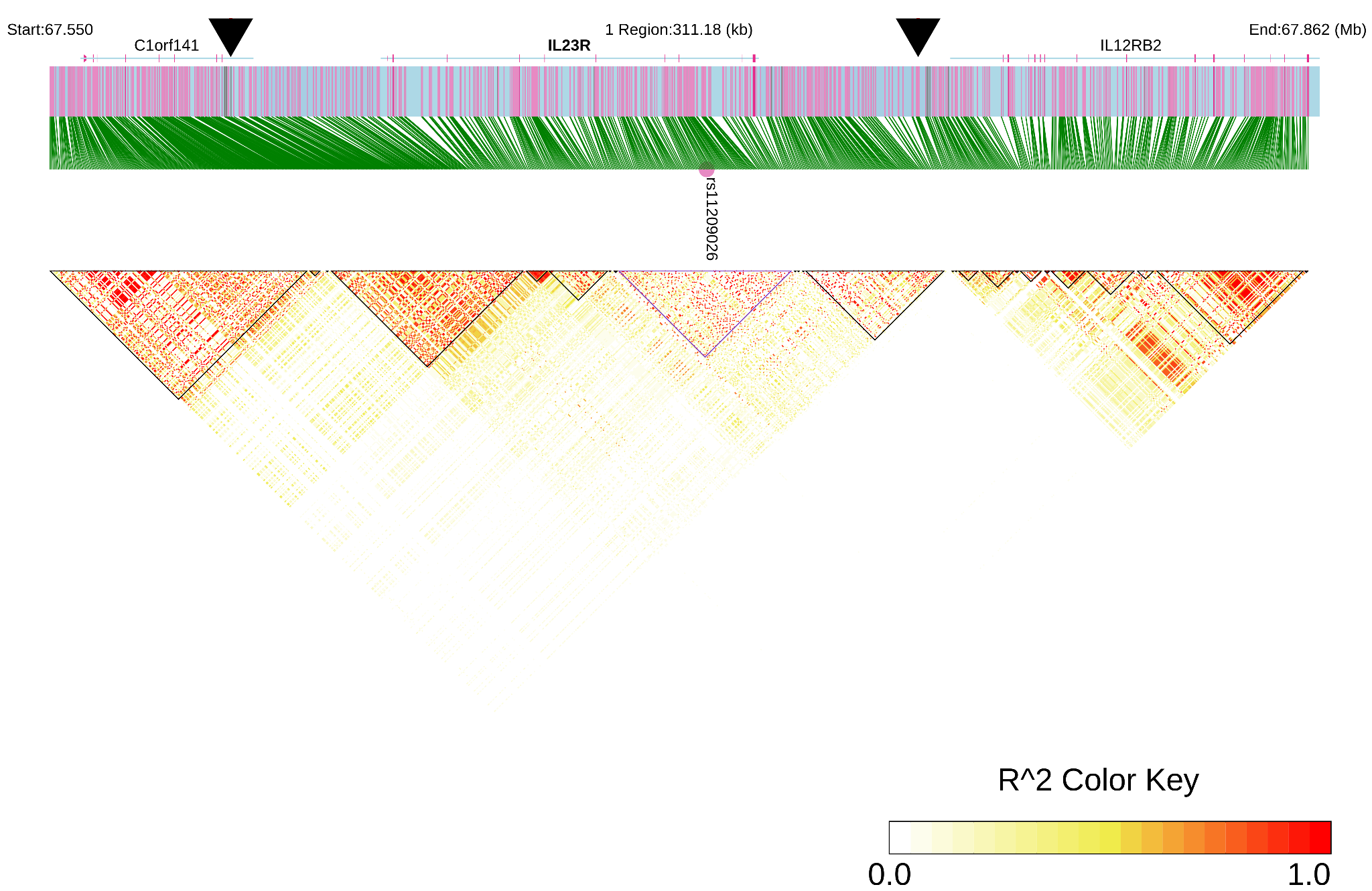
**

**Supplementary Figure S2.** Heatmap of linkage disequilibrium (LD) values (r^2^) between variants located in or in close proximity to *IL23R* in modern European populations using the 1000 Genomes Project (excluding the Finnish). The block where rs11209026 is located is highlighted by a purple triangle. Black arrows above the sequence track display recombination spots in Europeans according to HapMap Release 24.


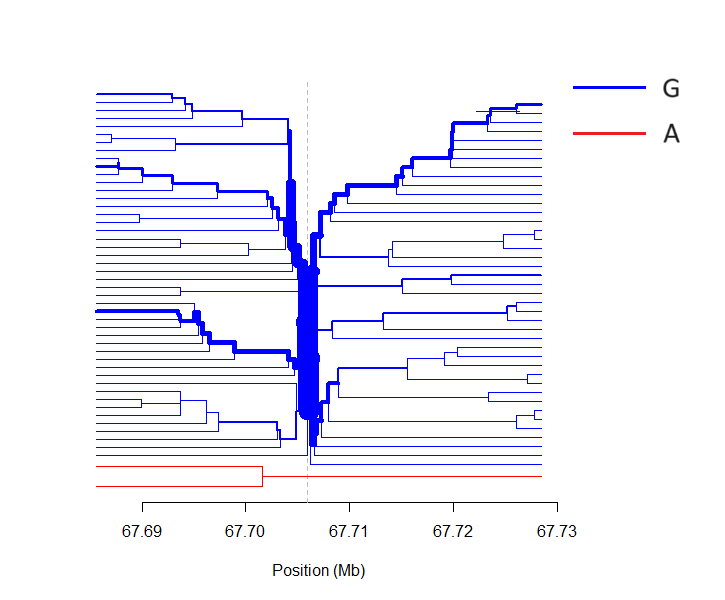


**Supplementary Figure S3.** Bifurcation tree for rs11209026 in CEU showing haplotypes with the A-allele (red) and G-allele (blue).


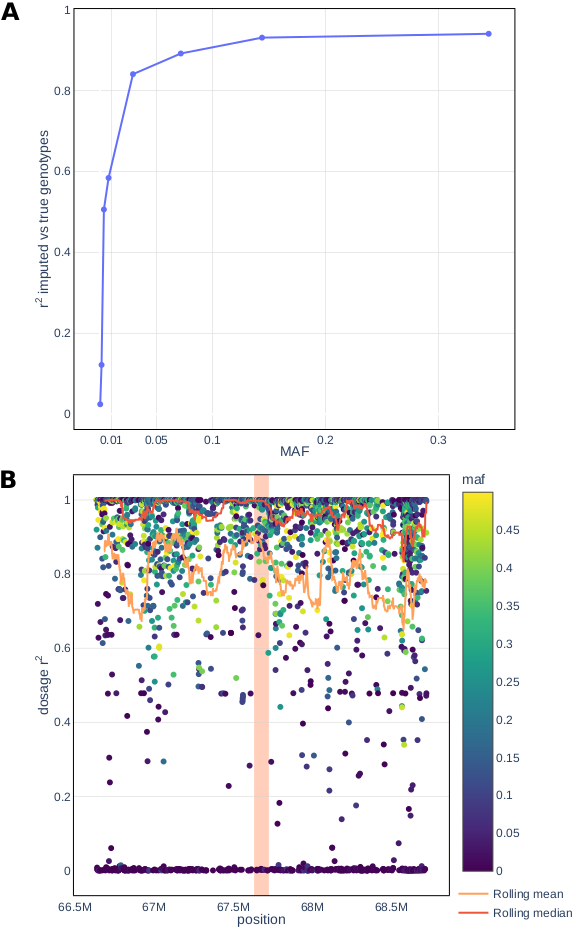


**Supplementary Figure S4.**  Validation of imputation of ancient genomes. **A.** Imputation accuracy (aggregated r^2^) as a function of minor allele frequency (MAF) for 24 high-coverage genomes (>20X) that were downsampled to 0.5X coverage. **B.** Imputation accuracy per variant (dosage r^2^). Highlighted in salmon is the region of the *IL23R* gene. The rolling mean and median were calculated using a window size of 100.


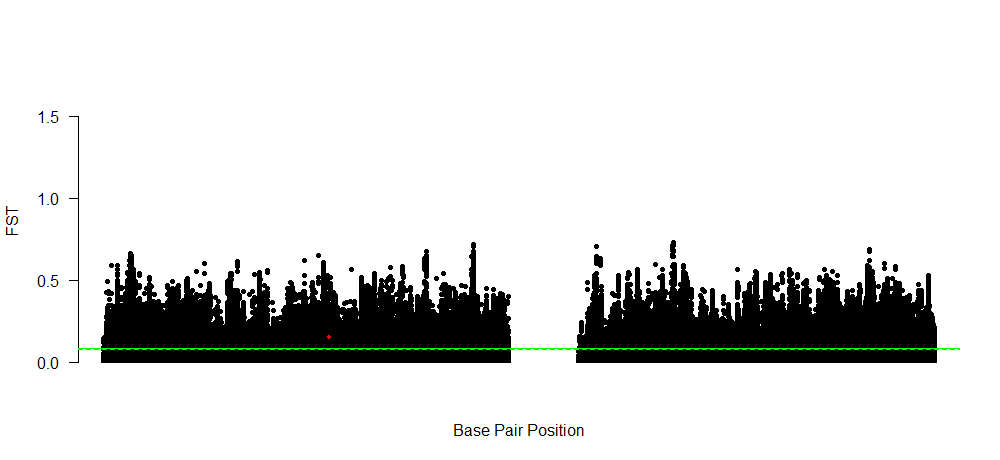


**Supplementary Figure S5.** Population differentiation (F_ST_) estimates between WHG and EF. The red dot represents rs11209026. The solid and dashed lines show the gene (*IL23R*) and the chromosome-wide F_ST_, respectively.


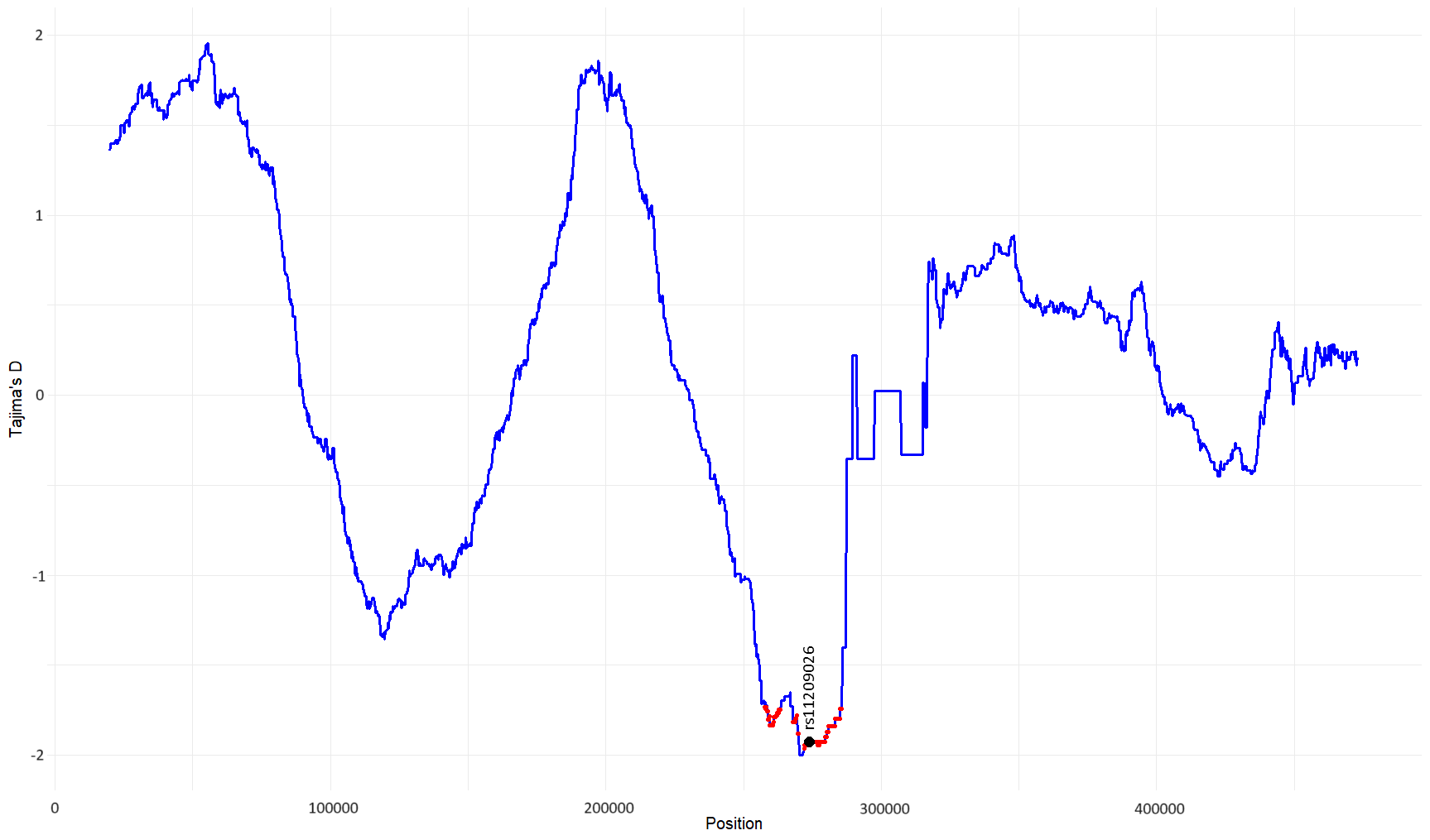


**Supplementary Figure S6.** Tajima`s D values for rs11209026-A haplotypes in a 500kb-region in CEU. The x-axis represents the position around the SNV while the y-axis shows the Tajima`s D values scale ranging from -2 to +2. Location of rs11209026 and regions that reach significance (p-value <0.05) (red) are shown.
