# Supplementary material for: Neolithic introgression of IL23R-related protection against chronic inflammatory bowel diseases in modern Europeans
Source: eBioMedicine. 2025 Feb 8;113:105591. doi: 10.1016/j.ebiom.2025.105591 (PMC11849592; doi:10.1016/j.ebiom.2025.105591)
Supplement: Supplementary Tables S3–S6 [file mmc4.docx]

Supplementary Materials for:

**Neolithic introgression of *IL23R*-related protection against chronic inflammatory bowel diseases in modern Europeans**

**Authors**

Ben Krause-Kyora^1^, Nicolas Antonio da Silva^1^, Elif Kaplan^1^, Daniel Kolbe^1^, Archaeological Civilization Disease Consortium (ACDC)^*^, Inken Wohlers^2^, Hauke Busch^3^, David Ellinghaus^1^, Amke Caliebe^4^, Efe Sezgin^5^, Almut Nebel^1^, Stefan Schreiber^1,6^

**This file includes:**

Supplementary Tables S3 – S6

**Supplementary Table S3.** Overview of the 66 Neolithic individuals merged with the AADR dataset.

| **Site** | **Grouping** | **Sample size** | **Dating (BCE)** | **Reference** |
| --- | --- | --- | --- | --- |
| Niedertiefenbach | Late Farmer | 25 | 3300 – 3200 | Immel et al. 2021 |
| Altendorf | Late Farmer | 7 | 3250 – 3100 | da Silva et al. 2023 |
| Warburg | Late Farmer | 9 | 3400 –2900 | da Silva et al. 2023 |
| Rimbeck | Late Farmer | 2 | 3300 – 2900 | da Silva et al. 2023 |
| Niederpöring | Early Farmer | 4 | 5200 – 4900 | da Silva et al. 2023 |
| Fellbach-Öffingen | Early Farmer | 10 | 5700 – 4900 | da Silva et al. 2023 |
| Trebur | Early Farmer | 9 | 5000 – 4500 | da Silva et al. 2023 |
| **TOTAL** |  | **66** |  |  |

**Supplementary Table S4.** Source for features and its coordinates (hg19) used to estimate putatively neutral regions.

| **Feature** | **Link** |
| --- | --- |
| Coding  sequences | http://hgdownload.cse.ucsc.edu/goldenPath/hg19/database/knownGene.txt.gz |
| Segmental  duplications | http://hgdownload.cse.ucsc.edu/goldenPath/hg19/database/genomicSuperDups.txt.gz |
| CNVs | http://dgv.tcag.ca/dgv/docs/GRCh37_hg19_variants_2020-02-25.txt |
| Conserved  elements | http://hgdownload.cse.ucsc.edu/goldenPath/hg19/database/phastConsElements46way.txt.gz |
| CpG islands | http://hgdownload.cse.ucsc.edu/goldenPath/hg19/database/cpgIslandExt.txt.gz |
| Repetitive  elements | http://hgdownload.cse.ucsc.edu/goldenPath/hg19/database/rmsk.txt.gz |
| Centromere  regions | http://hgdownload.cse.ucsc.edu/goldenPath/hg19/database/cytoBand.txt.gz |
|  |  |

**Supplementary Table** **S5.** Population differentiation (F_ST_) estimates between WHG and farmer populations comparing the mean F_ST_ of the chromosome, *IL23R* gene and rs11209026.

| **Populations** | **Mean F_ST_ of chromosome 1** | **Mean F_ST_ of *IL23R*** | **F_ST_ of rs11209026** |
| --- | --- | --- | --- |
| **WHG - AF** | 0.087 | 0.189 | 0.211 |
| **WHG - EF** | 0.078 | 0.082 | 0.153 |
| **WHG - LF** | 0.051 | 0.115 | 0.194 |
| **AF- EF** | 0.002 | 0.026 | -0.016 |
| **AF - LF** | 0.013 | 0.002 | -0.024 |
| **EF - LF** | 0.008 | 0.001 | -0.008 |

**Supplementary Table S6.** Hardy-Weinberg and Tajima`s D test results for rs11209026-G/A alleles for the Neolithic farmer and modern populations.

| Population | GG | GA | AA | Observed (HET_G) | Expected (HET_G) | P | Tajima`s D |
| --- | --- | --- | --- | --- | --- | --- | --- |
| Anatolian Farmers | 5 | 5 | 0 | 0.5 | 0.375 | 1 | 0.587 |
| Early Farmers | 21 | 8 | 1 | 0.266 | 0.278 | 1 | 0.888 |
| Late Farmers | 78 | 31 | 4 | 0.274 | 0.285 | 0.74 | 0.534 |
| CEU | 90 | 8 | 1 | 0.081 | 0.096 | 0.213 | 0.384 |
