# Supplementary material for: Neolithic introgression of IL23R-related protection against chronic inflammatory bowel diseases in modern Europeans
Source: eBioMedicine. 2025 Feb 8;113:105591. doi: 10.1016/j.ebiom.2025.105591 (PMC11849592; doi:10.1016/j.ebiom.2025.105591)
Supplement: Supplementary Data Affiliation of Consortium Collaborators [file mmc5.docx]

**Supplementary Data**

**Neolithic introgression of *IL23R*-related protection against chronic inflammatory bowel diseases in modern Europeans**

Ben Krause-Kyora, Nicolas Antonio da Silva, Elif Kaplan, Daniel Kolbe, Archaeological Civilization Disease Consortium (ACDC), Inken Wohlers, Hauke Busch, David Ellinghaus, Amke Caliebe, Efe Sezgin, Almut Nebel, Stefan Schreiber

**Archaeological Civilization Disease Consortium (ACDC) collaborators**

| Name | Affiliation |
| --- | --- |
| Sabine Schade-Lindig | Landesamt für Denkmalpflege Hessen, hessenARCHÄOLOGIE, Schloss Biebrich, Wiesbaden, Germany |
| Joachim Wahl | Institute for Archaeological Sciences, Palaeoanthropology Working Group, University of Tübingen, Tübingen, Germany |
| Carola Berszin | Anthropologische Dienstleistungen Konstanz, Konstanz, Germany |
| Michael Francken | Landesamt für Denkmalpflege im Regierungspräsidium Stuttgart, Konstanz, Germany |
| Irina Görner | Museumslandschaft Hessen Kassel, Sammlung Vor- und Frühgeschichte, Kassel, Germany |
| Kerstin Schierhold | LWL-Altertumskommission für Westfalen, Münster, Germany |
| Joachim Pechtl | Institut für Archäologie, University of Innsbruck, Innsbruck, Austria |
| Gisela Grupe | Biocenter of the Ludwig-Maximilians-Universität, München, Germany |
| Johannes Müller | Institute of Pre- and Protohistoric Archaeology, Kiel University, Kiel, Germany |
