# Supplementary material for: Neolithic introgression of IL23R-related protection against chronic inflammatory bowel diseases in modern Europeans
Source: eBioMedicine. 2025 Feb 8;113:105591. doi: 10.1016/j.ebiom.2025.105591 (PMC11849592; doi:10.1016/j.ebiom.2025.105591)
Supplement: Archaeological Civilization Disease Consortium [file mmc6.docx]

Archaeological Civilization Disease Consortium (ACDC) group members for PubMed indexing

| First names | Surname |
| --- | --- |
| Sabine | Schade-Lindig |
| Joachim | Wahl |
| Carola | Berszin |
| Michael | Francken |
| Irina | Görner |
| Kerstin | Schierhold |
| Joachim | Pechtl |
| Gisela | Grupe |
| Johannes | Müller |
